# The West London Hospital

**Published:** 1902-08-16

**Authors:** 


### THE YEAR'S WORK.

### THE WEST LONDON HOSPITAL.

### THE YEAR'S WORK.

The number of in-patients, from being 1,973 in 1900, increased in 1901 to 2,243. The number of available beds, including nine for isolation, was 133 in both years, and the increase is due to the fact that the average stay was reduced from 22·9 to 20·07. The cost per occupied bed was nearly £10 more than in the previous year; this is attributed to a variety of causes, among which are higher contract prices for provisions, increase in nursing and domestic staffs to do the larger amount of work necessitated by modern medical and surgical requirements, increased number of patients, and heavier demands for renewal of furniture, bedding, etc. There were 32,176 out-patients, an average of 267 daily, at a cost of 1s. 0¾d., or 4¼d. for each attendance.

The West London Post Graduate College had a successful year; 94 new names were enrolled, bringing up the total to 323. Thirty-six local practitioners have taken life-tickets. Altogether, 205 courses were taken out during the year. The ordinary income was £7,129 1s. 10d., extraordinary, £5,122 2s. The total ordinary expenditure was £10,145 2s. 3d.; extraordinary (repairs, building improvements, furniture and X-ray, and electric departments), £308 1s. 5d. King Edward's Fund gave £1,150, the Sunday Fund £600, and the Saturday Fund £259 19s. Invested property yielded £340 4s. 7d.

